# Recommendations of the Association of the Scientific Medical Societies in Germany’s (AWMF) Ad-hoc Commission Medical Devices on the handling of medical devices after explantation

**DOI:** 10.3205/000342

**Published:** 2025-07-11

**Authors:** Andreas Markewitz, Antje Aschendorff, Wolfram Mittelmeier, Boulos Asfour, Ansgar Berlis, Torsten Beyna, Jens-Uwe Blohmer, Mathias Gorenflo, Marcus Katoh, Wolfram Knapp, Thomas Lenarz, Folker Spitzenberger, Ludger Tüshaus, Peter M. Vogt, Gerald Werner, Mathias Wilhelmi

**Affiliations:** 1 Bendorf, Germany; 2 Department of Otorhinolaryngology, University Medical Center Freiburg, Germany; 3 Orthopaedic Clinic, University Medical Center Rostock, Germany; 4 Department of Pediatric Heart Surgery, University Medical Center Bonn, Germany; 5 Diagnostic and Interventional Neuroradiology, University Hospital Augsburg, Germany; 6 Medical Clinic, Evangelical Hospital, Düsseldorf, Germany; 7 Clinic for Gynecology with Breast Center of the Charité, Berlin, Germany; 8 Clinic for Pediatric Cardiology and Congenital Heart Defects, University Hospital Heidelberg, Germany; 9 Clinic for Diagnostic and Interventional Radiology, Helios Hospital Krefeld, Germany; 10 German Society for Nuclear Medicine, Göttingen, Germany; 11 Clinic for Ear, Nose and Throat Medicine, Hanover Medical School, Hanover, Germany; 12 Center for Regulatory Affairs in Biomedical Sciences, Lübeck University of Applied Sciences and Arts, Lübeck University of Technology, Lübeck, Germany; 13 Clinic for Pediatric Surgery at the University Medical Center Schleswig-Holstein, Campus Lübeck, Germany; 14 Clinic for Plastic, Aesthetic, Hand and Reconstructive Surgery, Hanover Medical School, Hanover, Germany; 15 University Heart and Vascular Center Frankfurt/Main, Germany; 16 Clinic for Vascular Surgery, St. Bernward Hospital, Hildesheim, Germany

**Keywords:** medical devices, explantation, legal requirements

## Abstract

The explantation of medical devices is a common procedure. However, the legal regulations and the resulting obligations of treating physicians and institutions regarding the handling of explanted medical devices are not always well known. The present recommendations aim to summarize the essential legal and practical aspects involved in the process following the explantation of medical devices.

## Abstract (de)

Die Explantation von Medizinprodukten ist ein häufiger Eingriff. Die gesetzlichen Vorschriften und die sich daraus ergebenden Pflichten der behandelnden Ärzte und Institutionen zum Umgang mit den explantierten Medizinprodukten sind jedoch nicht überall bekannt. Die vorliegenden Empfehlungen sollen die wesentlichen gesetzlichen und praktischen Aspekte beim Vorgehen nach Explantation von Medizinprodukten zusammenfassend darstellen.

## 1 General considerations

These recommendations contain suggestions for the procedure after removal of medical implants from the patient’s body. 

An implant becomes the property of the patient after insertion into the human body (§ 90, § 947, para. 2, BGB). After its removal, it remains the property of the patient. Usually, it is at the patient’s discretion what happens to the implant after explantation (explant): taking it home or disposal.

In the event of a suspected damage of the explanted device for which the manufacturer is responsible, the treating institution must immediately report this possible damage to the Federal Institute for Drugs and Medical Devices (BfArM) [1], [2], [3].

The legal basis for this reporting obligation is Section 3 of the Medical Devices User Reporting and Information Regulation (MPAMIV) [4].

The duty to report via the BfArM online portal applies to all persons who use medical devices professionally. This also applies if the user has informed the Institution’s Medical Device Officer, the institution’s internal medical technology department or the manufacturer of the device in question. 

At the same time, the explant must be appropriately examined if a serious incident is suspected. Requirements for documentation, testing and expertise of the expert must result in a reproducible assessment of the cause of damage. 

The examination methodology and the examination result must be documented as a supplement to the preoperative documentation of the implant function or implant dysfunction and must also be added to the medical record.

In the event of a suspected device failure, as a minimum measure, photographic documentation of the explant or the explant parts should be carried out by the explanting institution and kept as part of the medical record. 

The procedure for handling explants, in particular their destructive examination or the discarding of the explant, was previously regulated by the Medical Devices Safety Plan Regulation (MPSV), which expired on May 26, 2021, but is still frequently cited in this context. Since May 2021, however, Section 72 of the Medical Device Law Implementation Act (MPDG) [5] applies. According to this, “products and sample materials suspected of being involved in a serious incident may not be discarded until the risk assessment of the competent federal authority has been completed.” However, the transfer of the medical device to a legal or natural person other than the patient, e.g. to the BfArM as the competent federal authority, requires the patient’s documented consent. For the perioperative handling of explants, there should therefore be an internal hospital procedure or standard operating procedure (SOP) that takes into account the following aspects, without this list claiming to be exhaustive:

1. procedures for suspected or obvious damage events or product defects,
2. procedures for preserving the explanted medical device,
3. procedures for handing over a potentially defective explanted medical device to third parties for further analysis.

Further aspects on this topic were published by the Association of the Scientific Medical Societies in Germany’s (AWMF) ad hoc commission “Evaluation of medical devices” in June 2018 [6]. Other professional societies have also published information on this topic [7], [8], [9], [10].

## 2 Procedure

Before each explantation, the patient should not only be informed about the procedure in question, but also about the planned handling of the explant and the main reasons for the planned approach, which usually requires two different information sheets, each of which the patient must sign. The importance of the explant as possible evidence in the event of damage must be explained. An example based on a sample provided by the German Medical Technology Association (BVMed) on its homepage [11] is attached as Attachment 1 to this paper. The legal obligation to report suspected damage to the explant for which the manufacturer is responsible has already been mentioned. In such cases, it should also be noted that, although storage of the explant is not required by law, it is sensible for obvious reasons to do so in the view of the authors until any legal clarification has been completed. A reference to a lack of storage capacity is unlikely to be sufficient reason for most courts to discard the explant prematurely. 

If there is no suspicion of a reportable damage, the patient must decide and confirm with his signature

1. whether he/she wishes to have the explant handed over,
2. whether he/she wishes the explant to be discarded, or
3. whether he/she agrees to transfer ownership of the explanted device to the treating institution, e.g. for teaching and training purposes.

It should be noted that explants are no longer medical devices, but clinical waste according to the waste codes AS 180102, 180104 or, in the case of an infected explant, 180103 [12]. For aesthetic reasons and to minimize the potential risk of infection for the patient and their environment, explanted devices should be cleaned, disinfected and packaged liquid-tight before being given to the patient.

## 3 Individual steps in detail

Aspects for the informed consent discussion:

The information about the procedure and the planned handling of the explant are documented on information sheets and in the patient data management system (PDMS), if available.

For reasons of hygiene and due to the fact that the explant cannot be guaranteed to be free of infection, patients should be advised not to take the explant with them during the information session. This information about the risk of infection from explants must be included in the preoperative information sheets. It is useful to point out that a reliable assessment of the risk of infection is only possible after receipt of the histology and microbiology results, usually after 3 weeks.

If the transfer of the explant from the explanting facility to a third party, e.g. the manufacturer, is considered, the patient’s consent to the transfer should be explicitly noted. In this context, it seems justified to point out that the objectivity of the explant examination by the manufacturer is sometimes questioned in court proceedings.

### Cleaning and packaging

Smaller explants (screws, bolts, etc.) are cleaned at the operating table with rinsing solution. The surgical float cleans the explants with tap water. The blood is washed off and non-adherent tissue is removed. In the case of orthopaedic endoprostheses, small pieces of bone adhering to the surface are a possible aspect for an explant analysis and should not be removed. These arthroplasty explants (hip, knee, shoulder) are first placed in a 3% hydrogen peroxide solution and then rinsed. Cavities do not need to be rinsed. The explants are wrapped in absorbent material (e.g. Molinea^®^ disposable patient pads) and packed in zip bags.

The patient sticker and a sticker with the words “Caution! Risk of injury. Contains potentially infectious material. The institution accepts no liability!” are affixed. The patient is requested not to open the packaging inside the hospital.

If a patient does not wish to have their explant handed over, it will be disposed of appropriately – taking into account the above-mentioned exception of suspected damage or dysfunction of the explant and the resulting restrictions due to the preservation that should be carried out in these cases (see above) – unless it is required for further internal institutional measures such as training. 

### Handover to the patient

The explant is handed over to the patient as soon as possible. It can be handed over directly from the operating theater or sent to the ward. The handover of the explant to the patient is noted in the patient documentation, e.g. in the nursing report. If the patient requests delivery of the explant in deviation from their preoperative decision, this must be documented with the patient’s signature, e.g. on the preoperatively signed information sheet for explants.

### Suspected infection

If infection is suspected, explants should be sent to an institute for medical microbiology with the necessary microbiological request form. Large explants (e.g. total endoprostheses (TEP), plates, intramedullary nails) that are microbiologically examined can then be returned to the patient after reprocessing. For a microbiological examination, the explants usually have to be sent to the microbiology department in an implant box filled with Ringer’s solution; if there are any uncertainties in this regard, the relevant institute should be consulted as to how the shipment should be carried out. 

To enable the implant box and the explant to be returned, a delivery note or a comparable document must usually be sent along. It must be noted on the delivery note whether the patient wishes to receive the explant back. The explant will then be sent to the patient on the ward as soon as the examinations have been completed (usually 2–3 working days after receipt of the sample).

Small explants (e.g. screws, wires) that are examined microbiologically cannot be sent back to the patient after reprocessing. During the reprocessing process, the explants come into contact with reagents of animal origin. Despite subsequent autoclaving, freedom from prions cannot be guaranteed. It is therefore not possible to give them to patients due to the unpredictable risk of infection.

## Notes

### Disclaimer

The contents of these recommendations have been compiled with the greatest care. Nevertheless, no liability can be accepted for the completeness, accuracy and up-to-dateness of the information. Furthermore, the contents are recommendations and do not replace legal advice.

### Competing interests

The authors declare that they have no competing interests.

## Supplementary Material

Example of a patient information form
