# Supplementary material for: Recommendations of the Association of the Scientific Medical Societies in Germany’s (AWMF) Ad-hoc Commission Medical Devices on the handling of medical devices after explantation
Source: Ger Med Sci. 2025 Jul 11;23:Doc06. doi: 10.3205/000342 (PMC12372263; doi:10.3205/000342)
Supplement: Example of a patient information form [file GMS-23-06-s-001.pdf]

# Attachment 1: Example of a patient information form

## Declaration of consent to hand over a medical device for examination purposes

Dear patient, dear parents and caregivers,

You or your child or the person you are caring for have received a medical device. Your doctor has recommended that you replace the product or has already replaced it. We would like to inform you below about the technical examination of the product and ask for your support.

In the interest of patient safety, medical devices are regularly checked by the manufacturer and evaluated for possible risks according to strict standards. For this purpose, the medical device you have been using up to now should be examined. Such an examination is carried out in accordance with suitable test procedures. The examination is usually carried out by the manufacturer, as there are virtually no independent examination centers.

According to legal requirements, products that are the property of the patient may only be handed over for testing purposes with the prior consent of the patient or their legal or legal representative. This consent is also necessary if the product may be destroyed during the technical examination.

We therefore ask you to consent to the handover of the product and the subsequent technical examination. In this case, your doctor will hand over the product to the examination center for examination. You will receive a copy of the signed declaration of consent from your doctor for your records. You can also withdraw your consent at any time.

For reasons of hygiene and to minimize any risk of infection from the explant, we advise you not to take the explant with you. Should you nevertheless decide to have the explant handed over to you, we would like to point out that a reliable assessment of the risk of infection is only possible after receipt of the histological and microbiological results, usually after 3 weeks.

In the event that the product is destroyed during the examination, you have the right to receive photographic documentation of the condition of the product prior to the examination and a copy of the examination report. If you wish to do so, please contact the address given in the declaration of consent.

## Information on data protection:

In order to carry out the technical examination and to be able to provide you with photo documentation and an examination report on request, your personal data, including health data, and the data of your child or the person you are caring for (e.g. name, address, date of birth, date of explantation) will be passed on to the manufacturer and, where applicable, to its authorized sales company and processed.

This personal data will only be processed for the purposes listed in this form. The legal basis for data processing is your consent to the disclosure for examination purposes in conjunction with Section 72 (6) of the Medical Devices Law Implementation Act (MPDG). After the purpose has been achieved (performance of the examination and, if applicable, delivery of the examination report), the personal data or health data will be deleted, unless the manufacturer is entitled or obliged to further storage and processing required in the respective context on the basis of a legal authorization or legitimate interests.

Your consent to data processing can be withdrawn at any time without giving reasons and with effect for the future by contacting the manufacturer at its contact address. The withdrawal has no influence on the legality of the data processing that has taken place on the basis of the consent up to the time of withdrawal.

If you have any further questions about data processing or your rights as a data subject, you can also contact the data protection officer at the manufacturer's contact address.

---

________________________________________

Patient [surname, first name]

____________________

Date of birth

In the event that the patient is unable to give informed consent, consent must be given by the patient's legal or legally authorized representative. In this case, please also enter the name of the legal or legally authorized representative who is signing the declaration of consent:

________________________________________

Legal or legal representative [surname, first name]

________________________________________

Manufacturer of the medical device, if applicable authorized sales company

________________________________________

Contact address of the manufacturer

________________________________________

Product name [name, model]

______________________________

Item number

_______________________________________
LOT number / serial number

______________________________________________________________
Clinic / attending physician

_______________________________________
Date of explantation

Additional information

______________________________________________________________

______________________________________________________________

**I hereby consent to the handover of the above-mentioned medical device to the manufacturer, the competent higher federal authority (Federal Institute for Drugs and Medical Devices (BfArM)) or the experts commissioned by it for a technical examination.**

**At the same time, I relieve the attending physicians and medical personnel from their duty of confidentiality to the extent necessary for the handover of the product and to the extent to which I have consented to the handover.**

**I have been informed that the product may be destroyed during the examination. I am aware that in this case I can request photographic documentation of the condition of the product prior to the examination and a copy of the examination report from the manufacturer's contact address given above.**

**I have been informed that for reasons of hygiene and to minimize any risk of infection from the explant, the treating physician's advice against taking the explant with me, since a reliable assessment of the risk of infection is only possible after receipt of the histological and/or microbiological results, usually after 3 weeks.**

**I am aware that I will receive a copy of the signed declaration of consent from my doctor.**

Place, date: ____________________________________

____________________________________________
Signature of the patient or the legal or legally authorized representative

Attachment 1 to: *Markewitz A, Aschendorff A, Mittelmeier W, Asfour B, Berlis A, Beyna T, Blohmer JU, Gorenflo M, Katoh M, Knapp W, Lenarz T, Spitzenberger F, Tüshaus L, Vogt PM, Werner G, Wilhelmi M. Recommendations of the Association of the Scientific Medical Societies in Germany's (AWMF) Ad-hoc Commission Medical Devices on the handling of medical devices after explantation. GMS Ger Med Sci. 2025;23:Doc06. DOI: 10.3205/000342, URN: urn:nbn:de:0183-0003423*
